# Pharmacological Implications of Adjusting Abnormal Fear Memory: Towards the Treatment of Post-Traumatic Stress Disorder

**DOI:** 10.3390/ph15070788

**Published:** 2022-06-24

**Authors:** Chen-Cheng Lin, Yia-Ping Liu

**Affiliations:** 1 Laboratory of Cognitive Neuroscience, Department of Physiology and Biophysics, National Defense Medical Center, Taipei 114, Taiwan; wsadhjkl@gmail.com; 2 Department of Psychiatry, Cheng Hsin General Hospital, Taipei 112, Taiwan; 3 Genomics Research Center, Academia Sinica, Taipei 115, Taiwan; 4 Department of Psychiatry, Tri-Service General Hospital, Taipei 114, Taiwan

**Keywords:** fear memory, post-traumatic stress disorder, pharmacological therapy, monoamines, glucocorticoid receptors

## Abstract

Post-traumatic stress disorder (PTSD) is a unique clinical mental abnormality presenting a cluster of symptoms in which patients primarily experience flashbacks, nightmares and uncontrollable thoughts about the event that triggered their PTSD. Patients with PTSD may also have comorbid depression and anxiety in an intractable and long-term course, which makes establishing a comprehensive treatment plan difficult and complicated. The present article reviews current pharmacological manipulations for adjusting abnormal fear memory. The roles of the central monoaminergic systems (including serotonin, norepinephrine and dopamine) within the fear circuit areas and the involvement of the hypothalamic-pituitary-adrenal (HPA) axis and glucocorticoid receptor (GR) are explored based on attempts to integrate current clinical and preclinical basic studies. In this review, we explain how these therapeutic paradigms function based on their connections to stages of the abnormal fear memory process from condition to extinction. This may provide useful translational interpretations for clinicians to manage PTSD.

## 1. Current Treatment Regime of PTSD

How individuals react to traumatic experiences is an old topic; however, post-traumatic stress disorder (PTSD) is a relatively new diagnosis in clinical psychiatry. The name PTSD was initially presented in the *Diagnostic and Statistical Manual of Mental Disorders, Third Edition* (DSM-III) by the American Psychiatric Association (APA) in 1980, in an effort to reintroduce war neurosis into official nomenclature [1]. Since then, the disorder has been widely recognized and investigated far beyond the description of war neurosis. The impacts of traumatic events are not easily resolved; individuals may exhibit a variety of psychological dysfunctions including anxiety, depression, behavioral hyperarousal and intrusive recollections of the traumatic experience even after they are safe [1,2,3]. Although these symptoms are indicative of PTSD, fear-related memory abnormality is considered the primary psychopathology [4,5]. It reflects the ultimate goal of human evolution to dispose of fear memories.

Treatments available for PTSD are very diverse. Psychological therapy and pharmacological regimes are two in the mainstream, and they can be applied conjointly. For pharmacological interventions, agents that restore synaptic concentrations of central monoamines, including selective serotonin reuptake inhibitors (SSRIs) and serotonin-norepinephrine reuptake inhibitors (SNRIs), are firstly considered. Agents that regulate adrenergic activity through the beta receptors are also used to manage short-term anxiety symptoms of PTSD.

## 2. Current Challenges of Managing PTSD

Restoration of patients’ cognitive function is the main strategy in treating PTSD; however, the real-world efficacy of pharmacological treatment is far from adequate, causing PTSD to be among the most difficult mental disorders to manage. For example, neurochemical agents prescribed to treat the symptoms of PTSD primarily target the receptors of monoaminergic systems, presenting favorable outcomes in relieving depression and anxiety; however, most of them do not effectively treat the dysfunction of extinction retrieval, the underlying pathogenesis of PTSD, to resolve the impairment of fear memory processing. Apparently, there is a gap in knowledge between the extent of pharmacological agents and the underlying mechanisms of PTSD. 

In this review, in a convergent manner, we integrate clinical and preclinical evidence to provide explanations of the therapeutic mechanisms of PTSD, particularly focusing on their connections to the impairment of fear memory processing.

## 3. Therapeutic Mechanisms of Pharmacological Treatments

### 3.1. Targeting Serotonergic Systems

A meta-analysis study in clinical psychiatry recommends three types of SSRIs, namely, fluoxetine, paroxetine and sertraline, as the first-line medications for patients with PTSD [6,7]. SSRIs have been broadly employed for many years to treat mood-related disorders. Because the brain regions constituting fear circuits (i.e., prefrontal cortex, hippocampus, and amygdala) have abundant 5-hydroxytryptamine (5-HT) receptors, central 5-HT systems are thus highly responsible for PTSD-induced mood changes. This is helpful for reducing the occurrence of depressive and anxiety symptoms after traumatic events [8,9]. 

Depression is a heterogeneous psychiatric disorder and is co-morbid with many other mental illnesses, including PTSD. Approximately 48% to 55% of patients with PTSD currently experience depression, or have had it in the past [10]. Because there is such a high rate of comorbidity, the chances are that SSRIs only correct PTSD-associated depressive moods, rather than fear symptoms. To examine this possibility, clarifying the relationship between depression and fear is necessary. Employing single prolonged stress (SPS) in a rodent model of PTSD, Lin et al. approached the symptoms of depression and fear separately [11]. They found that a 14-day sub-chronic regimen of escitalopram, an acknowledged SSRI, successfully restored SPS-induced depressive and anxiety symptoms (as indexed in the sucrose preference test and elevated T-maze), but not the SPS-impaired retrieval of fear extinction (as indexed in the Pavlovian fear conditioning paradigm). This evidence appears to support the notion that PTSD and depression exemplify distinct trauma-related phenotypes [12].

For an alternative interpretation, it is also highly possible that the central 5-HT system may serve as a common mechanism shared by both depression and fear. Therefore, adjusting the profiles of the central 5-HT system may facilitate the amelioration of fear memory after traumatic stress [13,14,15].

Increasing evidence has revealed that, in patients with PTSD, central 5-HT activity is downregulated in the fear circuit region of the brain, and both the binding affinity of 5-HT transporters (5-HTTs) and the density of 5-HT1B receptors are reduced in the amygdala and anterior cingulate cortex (ACC) [16,17,18]. Following this stream, 5-HT deficiency in the hippocampus enhances context-dependent fear conditioning, which could be prevented by intracerebroventricular 5-HT administration. This highlights the effects of central 5-HT in modulating hippocampus-dependent contextual fear conditioning [19,20]. For the amygdala, somatosensory information conveying conditioned stimuli [21]/unconditioned stimuli (US) connotations are mainly received by the lateral amygdala (LA), which is responsible for relaying information and transferring it to the basal amygdala (BA) [22], a region filled with 5-HT innervations projected from the dorsal raphe nucleus to accomplish crucial neuronal functions for conditioned learning. 

Although preclinical studies support the primary role of 5-HT in relieving fear memory, which is unsatisfactory in therapeutic use, some patients still experience their traumatic feelings often [23,24]. The inconsistency between clinical and preclinical evidence should be solved with a plausible interpretation regarding the dynamic optimization of area-dependent 5-HT functions. The therapeutic windows of SSRIs for depression and fear symptoms are possibly not unique; thus, the optimal dose of SSRIs for correcting trauma-induced depression is not the same as that for trauma-induced fear [25].

### 3.2. Targeting Noradrenergic Systems

Changes in noradrenaline (or norepinephrine, NE) activity in both the autonomic nervous system (ANS) and the central nervous system (CNS) contribute to the pathophysiology of PTSD [26,27,28,29]. The functional activity of the sympathetic nervous system in PTSD patients is enhanced [30,31,32,33]. In the CNS, the locus coeruleus (LC) is the principal site for the synthesis of norepinephrine; thus, the activity of the LC–NE system serves as a candidate in connecting the ANS and CNS to explain the hyper-arousal symptom of PTSD [27,34].

Preclinical studies have revealed that central NE involvement in fear conditioning and extinction is highly dependent on stress level and the targets of LC projection. When animals are under high levels of stress (e.g., foot shock), their LC–NE promotes aversive learning (i.e., fear conditioning) by enhancing the activity of the amygdala and inhibiting the activity of the medial prefrontal cortex (mPFC) through the involvement of α1 and β2 NE receptors [35,36]. This observation can be also interpreted by considering extinction a form of new learning that leads to deconditioning or extinction, in which stress impairs learning by activating the LC–NE and basolateral amygdala (BLA) [35,37]. When animals are under low levels of stress (e.g., cue-induced fear emotion), LC–NE promotes an extinguished aversive response (i.e., extinction learning) by removing downgraded inhibition from the mPFC to the amygdala through the β2 NE receptors [35]. This is consistent with clinical evidence that β2 NE receptors are highly accountable for PTSD in patients with childhood trauma [38]. 

The LC–NE projection to the hippocampus (via β receptors) is responsible for fear of a contextual nature. It enhances the consolidation of hippocampus-dependent contextual fear, but not the fear-associated specific cue [39,40,41]. Contextual conditioning is a psychological process in which background information is associated with a stimulus, leading to a given response. In this regard, fear generalization through aversive contextual processing prompts the development of PTSD [42]. Furthermore, within hippocampal formation, sub-regions receiving different monoaminergic projections are also responsible for context-dependent fear memory. For NE, the projection goes to the dentate gyrus [43], and for 5-HT, the dorsal hippocampus [20].

Evidence has revealed that epinephrine (EPI), the end-product of the NE biosynthetic pathway, is also a vital factor of PTSD occurrence in terms of contextual learning. For example, mice can be induced to freeze when they are contextually conditioned to fear, but this phenomenon does not show in mice without the enzyme transfer of norepinephrine to epinephrine (i.e., PNMT-KO mice). The peripheral administration of EPI and fenoterol (a selective β2-NE receptor agonist) effectively restore contextual traumatic memories in PNMT-KO mice, indicating that epinephrine strengthens contextual fear learning by acting in peripheral β2-NE receptors. Thus, epinephrine may play a causal role in the maintenance of contextual traumatic memories [44]. This illustrates that through either norepinephrine or epinephrine, β-NE receptors help to consolidate contextual traumatic memories, and once these traumatic memories are consolidated, extinction becomes difficult. 

As mentioned above, the employment of β antagonists such as propranolol are theoretically advantageous for the treatment of PTSD. Propranolol is easily transported across the blood–brain barrier, and exerts beneficial peripheral and central effects. Propranolol has been used extensively to relieve the peripheral symptoms of anxiety disorders [45]. Systematic meta-analyses have revealed that the timing of propranolol’s administration is crucial to its effectiveness. If administered immediately or shortly after a traumatic event, propranolol does not prevent the development of PTSD [46]. However, when administered long after the traumatic event, propranolol reduces not only the degree of physiological responses, such as the disturbance of heart rate, skin conductance and blood pressure in patients with PTSD during trauma memory reactivation, but also the severity of PTSD symptoms as measured through the Clinician-Administered PTSD Scale (CAPS) and PTSD Checklist Specific (PCL-S) [46,47]. That is, if PTSD has developed, propranolol ameliorates both the peripheral and central symptoms. Propranolol also affects the fear conditioning process in the fear circuit. When administered prior to the reactivation of a traumatic memory, propranolol resets dendritic spines in the BLA, the area responsible for cue-dependent conditioning, to the pre-trauma levels; however, it does not have this effect on the dorsal hippocampus, the area responsible for generalized contextual conditioning [48]. 

In clinical use, propranolol reduces both anxiety symptoms and fear memory-associated discomfort in patients with PTSD, which is far beyond the contribution of the β2 receptor in adjusting contextual fear conditioning [39,40,41]. Because propranolol is a nonselective β-blocker, the role of the β1 receptor must be clarified. Preclinical approaches support this possibility by showing that β1 antagonism in the BLA may reduce corticosterone-induced auditory-cue fear conditioning [49].

### 3.3. Targeting DA Systems

Similarly, the implications of peripheral and central NE are different in fear memory; the roles of peripheral and central DA are different too. Compared with central DA, peripheral DA is more easily obtained; thus, it has been considered for the evaluation of patients with PTSD. Yehuda and colleagues first demonstrated that the severity of PTSD, particularly the intrusion symptom, was correlated to augmented urinary DA levels in Vietnam combat veterans [29]. Costanzo and colleagues recruited veterans who served in Iraq or Afghanistan (representing a subthreshold population of combat-related PTSD) and examined their plasma DA and physiological reactions during fear conditioning. They found that during fear inhibition, a form of deconditioning processing, plasma DA had an inverse relationship with heart rate. This finding was specific to DA, but not epinephrine or norepinephrine [50]. These findings demonstrate that peripheral DA is associated with the fear conditioning process and is sensitive when the strength of fear processing is altered. 

For central DA, its pathway originates from the ventral tegmentum area (VTA) and is highly involved in the regulation of fear conditioning and extinction [51]. In terms of fear conditioning, Fadok and colleagues demonstrated that the restoration of DA synthesis within the VTA resolved impairments resulting from cue-dependent conditioning in DA-deficient mice [52]. Lin and colleagues showed that, during extinction, the reduction in efflux and the reuptake of DA in the amygdala were highly associated with the ineffectiveness of fear extinction retrieval in rats that experienced SPS [53]. These data suggest that strengthened central DA relieves the severity of PTSD because fear extinction is disrupted when the central DA function is restrained [54]; thus, as expected, DA repletion can recover DA depletion-impaired fear conditioning [52]. 

DA profiles in fear memory dysfunction are time-dependent and area-specific, and DA efflux levels are found to reduce specifically in the amygdala [53,55]. DA profiles are also time-dependent; their efflux in the amygdala increases immediately after acute stress [56], yet their responsiveness decreases as time goes by after the traumatic event. This deficit of resilience contributes to the ineffectiveness of fear memory extinction in PTSD [53]. Because the blockade of DA receptors sabotages fear extinction and does not impair fear acquisition during the conditioning stage [57], conditioning and extinction are not necessarily associated with each other.

Therefore, it appears that a weakened central DA function is associated with the ineffectiveness of extinction. Pharmacologically enhancing DA activity in key areas within the fear circuit may improve fear extinction, as observed in the mPFC [54,58]. Outside of the fear circuit, strengthened DA is beneficial in coping with stress. In the mesoaccumbal pathway, DA transmission is associated with various coping strategies for stressful events [59], whereas midbrain DA transmission is associated with resilience to stress [60]. In the mesolimbic pathway, DA transmission not only contributes to stimulus–reward learning but also encodes aversive prediction stimuli and prediction errors. This is beneficial for extinction learning, omitting the signaling of the expected aversive outcomes in a similar way to the mechanism of prolonged exposure therapy [61,62], one of the psychological treatments of PTSD.

Subtypes of DA receptors in a given area of a fear circuit also play important roles in regulating fear conditioning and extinction processes according to different signaling pathways. The activation of D1 receptors (D1R) strengthens the cAMP/PKA pathway, whereas the activation of D2 receptors (D2R), through the Gα protein of the i/o class (Gαi/o), inhibits adenylate cyclase. For example, infralimbic cortex (IL) D1R signaling contributes to the maintenance of fear conditioning by reducing the synaptic input from the IL to the amygdala [63], but the activation of IL D2R leads to the facilitation of fear extinction [57,64]. In the hippocampus and amygdala, D1R signaling primarily contributes to fear conditioning, whereas D2R signaling is responsible for fear extinction [65,66].

Recently, increasing evidence has demonstrated that the enzyme responsible for the degradation of DA plays a role in the top-down regulation of fear-related extinction learning, possibly through the change in the prefrontal DA level. Catechol-O-methyltransferase (COMT) Val158/108Met (rs4680) polymorphism is associated with fear inhibition and extinction deficits in PTSD. Lonsdorf and colleagues reported that Met/Met carriers exhibit a 40% reduction in COMT activity that leads to a heightened dopaminergic tone in the cortex and enhances fear memory consolidation or fear extinction resistance [67,68]. At first glance, the COMT result implies that an augmented DA transmission is therapeutically unfavorable, which is inconsistent with the notion that a robust DA function is beneficial for treating PTSD. However, as DA levels exhibit an inverted-U relationship with the function of the prefrontal cortex [69], too little or too much DA signaling is harmful for working memory. It appears that the optimization of central DA functions should be more appropriate to treat fear memory dysfunction in patients with PTSD. 

DA optimization was a challenge in clinical therapeutics until the emergence of aripiprazole, a third-generation antipsychotic acknowledged as a DA receptor modulator. Aripiprazole normalizes the imbalanced condition of central DA by its unique partial agonistic effect of D2R [70]. This unique characteristic is beneficial in correcting the ineffectiveness of fear extinction and is supported by pre-clinical rodent studies. Acute aripiprazole facilitates fear extinction through neuronal activation in the medial prefrontal cortex [71]. If administered a week after SPS (which is more similar to the development of PTSD because extinction ineffectiveness occurs over time), sub-chronic aripiprazole effectively reverses SPS-impaired fear memory dysfunction and SPS-reduced DA efflux in the amygdala [72].

### 3.4. Targeting the Hypothalamic-Pituitary-Adrenal Axis and Glucocorticoid Receptors

In general, the hypothalamic-pituitary-adrenal (HPA) axis helps to maintain homeostasis during many cortisol-related physiological functions. When the axis fails to exert its feedback regulation, individuals become physically and mentally abnormal. According to cortisol performance, the feedback dysfunction of the HPA axis varies in different mental disorders. For example, the cortisol level rises in people with depression, but falls in those with PTSD [73,74]. Yehuda et al. demonstrated that the manipulation of the HPA axis can serve as an adjunct method to exposure psychotherapy such as prolonged exposure (PE) [75].

The involvement of HPA axis dysregulation in PTSD is much greater than that of hormonal effects. Increasing evidence suggests that the enhancement of the activity of the central glucocorticoid receptor (GR) is responsible for the disturbance of HPA negative feedback in PTSD [74,76]. The GR helps to adapt stress and ensures that individuals maintain a balanced state [77,78,79]. When the GR is overly active, it augments fear conditioning, mirroring what happens in patients with PTSD. Specifically, GR expression in the fear circuit areas becomes enhanced [73,80,81], and the peripheral levels of plasma glucocorticoid are reduced in line with the negative feedback relationship of the HPA axis [82,83]. It appears that while employing GR manipulations to treat abnormal fear memory, two factors need to be considered: (i) the choice of appropriate GR-manipulating agents, and (ii) the time of administering these agents to the patients [84,85,86,87].

In a pilot clinical study, mifepristone (a GR antagonist known as RU486) in a short-term and high-dose regimen (i.e., 600 mg for 7 days) effectively increased the plasma levels of cortisol and the adrenocorticotropic hormone and reduced the severity of PTSD symptoms, and the behavioral improvements could last for 3 weeks after the end of the pharmacological regimen [86]. Furthermore, the responding sensitivity of the GR is particularly vital to regulating cortisol levels. Patients with a higher pretreatment sensitivity exhibited greater improvement when challenged with hydrocortisone [75]. The underlying mechanism remains unsolved, yet increasing evidence suggests that this GR-associated fear conditioning is highly dependent on the balance between FKBP prolyl isomerase 4 (FKBP4) and FKBP prolyl isomerase 5 (FKBP5). FKBP4 acts as an activated factor that leads the GR to translocate to the nucleus, and FKBP5 serves as an inhibitory chaperone that prevents GR translocation to the nucleus.

Along the GR signaling pathway, FKBP4 and FKBP5 operate at the upper stream before transcription, whereas the extracellular signal-regulated kinase (ERK, belonging to the family of mitogen-activated protein kinase, MAPK) and early growth response protein 1 (EGR1) serve as downstream elements after GR transcription. GR-activated fear conditioning in fear circuit areas is often combined with an increase in MAPK and EGR1. Enhanced fear memory is associated with GR over-expression and FKBP5 dysregulation with an increase in ERK1 and ERK2 phosphorylation and EGR1 expression in the hippocampus and amygdala [88,89,90]. This helps to explain the mechanism of using a GR antagonist, such as mifepristone, to treat patients with PTSD. However, it is intriguing that agents with GR agonistic characteristics, such as hydrocortisone or dexamethasone, appear helpful too. They increase peripheral cortisol levels and regulate cortisol-associated biological stress to reduce the incidence of PTSD [91,92]. GR agonists are also beneficial for fear extinction in patients with PTSD through adjusting GR and FKBP5 functions in the hippocampus and amygdala [93,94].

Following the above, we conclude that both GR agonists and antagonists can be beneficial in correcting abnormal fear memory, depending on the stage of development of the dysfunction. GR agonists facilitate fear extinction [94,95,96], whereas GR antagonists inhibit the consolidation of fear conditioning [86,89]. This is largely dependent on the function of FKBP5, which acts as an inhibitory chaperone, preventing GR translocation to the nucleus [97]. GR agonists, such as dexamethasone, can trigger FKBP5 synthesis to diminish GR activity [94,98], which enhances extinction learning [94]. GR antagonists such as mifepristone, however, directly suppress GR transcription and reduce the synthesis of FKBP5, ERK, and EGR1, which leads to difficulty in consolidating fear memory [84,85,86,89].

Regarding the timing of the clinical intervention in terms of GR agonists, a placebo-controlled double-blind study demonstrated that administering dexamethasone the night before the fear conditioning paradigm improved extinction by correcting the safety discrimination deficits in patients with PTSD [95]. In another placebo-controlled double-blind study, dexamethasone treatment combined with trauma memory reactivation tasks successfully reduced PTSD symptoms [96]. These studies support GR agonists’ facilitation of fear extinction and justify the combination of dexamethasone and exposure psychotherapy. Time-dependent effects on the conditioning–extinction processes of GR manipulation were also observed in preclinical studies. When administered immediately following fear conditioning, corticosterone facilitated stimulus generalization and prompted fear activity in which the dental gyrus was highly involved [99,100]. However, if administered prior to extinction learning, corticosterone facilitated the extinction process through adjusting the connectivity between the hippocampus and the ventromedial PFC [101]

## 4. Conclusions

**Figure 1:**
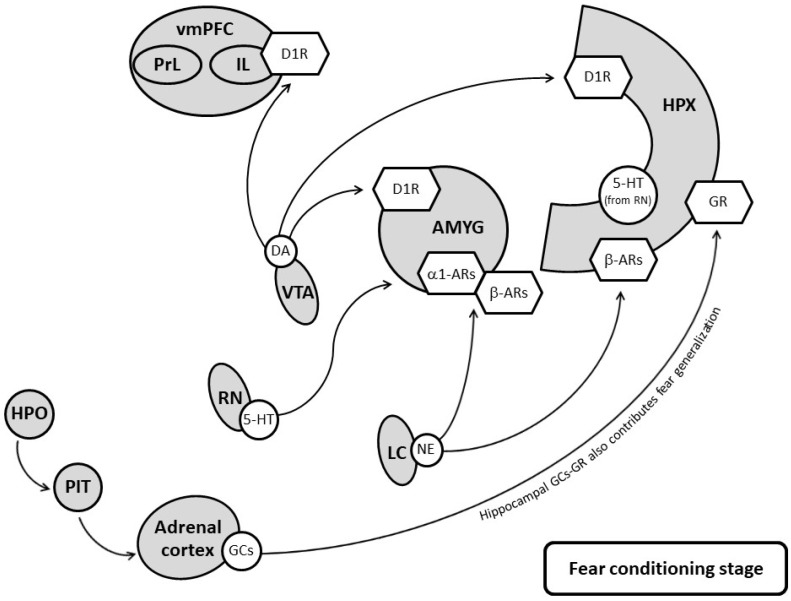
The regulations of fear conditioning by neurochemicals in the fear circuit. Monoamines and glucocorticoids contribute to fear conditioning through their corresponding receptors. vmPFC: ventral medial prefrontal cortex; IL: infralimbic cortex; PrL: prelimbic cortex; HPX: hippocampus; AMYG: amygdala; VTA: ventral tegmentum area; LC: locus coeruleus; RN: raphe nucleus; HPO: hypothalamus; PIT: pituitary; 5-HT: serotonin; NA: noradrenaline; α1-Ars: α1 NA receptors; β-Ars: βNA receptors; DA: dopamine; D1R: D1 receptors; GCs: glucocorticoids; GR: glucocorticoid receptor. The solid line refers to the strengthening of fear conditioning.

**Figure 2:**
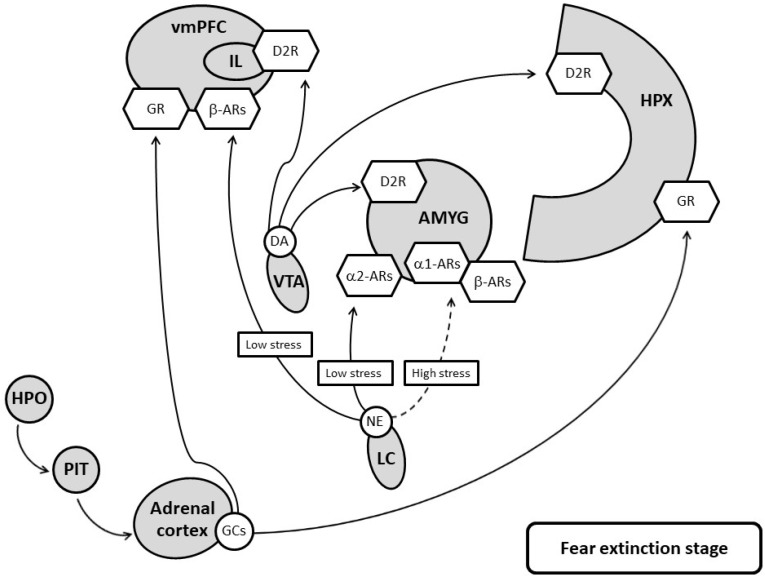
The regulations of fear extinction by neurochemicals in the fear circuit. DA, NE and glucocorticoids contribute to fear deconditioning through their corresponding receptors, but NE disrupts fear deconditioning when under extreme stress. vmPFC: ventral medial prefrontal cortex; IL: infralimbic cortex; HPX: hippocampus; AMYG: amygdala; VTA: ventral tegmentum area; LC: locus coeruleus; RN: raphe nucleus; HPO: hypothalamus; PIT: pituitary; NA: noradrenaline; α1-ARs: α1 NA receptors; α2-ARs: α2 NA receptors; β-ARs: βNA receptors; DA: dopamine; D1R: D1 receptors; D2R: D2 receptors; GCs: glucocorticoids; GR: glucocorticoid receptor. The solid line refers to the strengthening of fear extinction, the dashed line refers to the weakening of fear extinction.

Current therapeutic strategies target various aspects of PTSD. Pharmacological manipulations of monoaminergic transmission and glucocorticoid receptors should focus on the targeted brain areas and the timing of conditioning and the extinction of fear memory (for an illustration, see Figure 1 and Figure 2). For 5-HT, SSRIs are not beneficial for treating the fear-associated symptoms of PTSD if they are administered at dosages that effectively manage depressive disorders. For NE, β receptors help consolidate contextual traumatic memory, and when the traumatic memories are consolidated, they are difficult to remove. Β antagonists are unable to prevent PTSD, but they assist in the treatment of the disease after it has developed. For DA, the loss of resilience contributes to the ineffectiveness of fear memory extinction in patients with PTSD, and the optimization of the central DA function is therefore more appropriate. Finally, the abnormalities of the HPA axis also contribute to the development of PTSD across different stages. Corticosterone facilitates fear generalization and fear extinction, depending on the intervention time.

## Data Availability

This review did not create or analyze new data. Data sharing is not applicable to this review.
